# Exploring the use of clay pots as sustainable storage containers to improve water quality

**DOI:** 10.1186/s42506-024-00164-w

**Published:** 2024-07-15

**Authors:** Alaa K. Ibrahim, Ghada Said, Mai M. Badr

**Affiliations:** 1 Department of Environmental Health High Institute of Public Health, Alexandria University, 165 El-Horreya Avenue, El-Ibrahimia, Alexandria, Egypt; 2 Environmental Chemistry and Biology, Department of Environmental Health, High Institute of Public Health, Alexandria University, 165 El-Horreya Avenue, El-Ibrahimia, Alexandria, Egypt

**Keywords:** Water quality, Clay pot, *Legionella*, CaCO_3_, Treatment

## Abstract

**Background:**

Currently, tap water consumption is not highly preferred in Egypt and around the world. People prefer to consume bottled water because they believe that it is much safer and tastes better than tap water. Unfortunately, this preference can create an economic burden for many people, especially in developing countries. Clay pots can be used to provide cool, alkaline drinking water because of their porous micro-texture, which traps pollutants. This study aimed to investigate the use of clay pots to store tap water and its impact on the requirements for drinking water quality. This is done with the intent to decrease the need for bottled water as a means of offering a more sustainable and economical option.

**Methods:**

In this study, the efficiency of clay pots as sustainable storage containers for drinking water was tested by measuring physicochemical parameters (pH, TDS, EC, turbidity, DO, ammonia, chloride, total hardness, Ca hardness, Mg hardness, chlorine, Zn, and CaCO_3_) and biological parameters (TPC and *Legionella*).

**Results:**

After 7 days of storage, the quality of the water stored in clay pots met the standards set by the Egyptian law with a significant difference (*p* < 0.05) before and after the storage of water It was found that the dissolved oxygen increased from 6.17 ppm to 7.52 ppm after 7 days. As for total hardness, it declined from 195 to 178 ppm. There was also a significant drop in terms of TDS from 338 to 275 ppm. Furthermore, clay pots effectively filtered out both total viable bacteria and *Legionella*.

**Conclusion:**

This study proved the efficiency of using these containers with respect to some indicator values for tap water and tank water analysis. Clay pots are an excellent, cost-effective, and sustainable alternative for storing water.

## Introduction

Safe access to drinking water is an essential human right. Poor sanitation and deterioration of drinking water are the main causes of transmission of many diseases, such as cholera, diarrhea, and dysentery [1]. As water from surface water resources enters treatment plants, it is crucial to make it safe for drinking. Chlorination is the most common method of water disinfection in these treatment plants. It is a method used worldwide for reducing epidemic diseases. Drinking water should also be free of color, turbidity, odor, and microbes [2].

Unfortunately, drinking water quality in many developing countries is constantly compromised due to high population growth, industrial development, and the dumping of wastewater and chemical effluents into canals and other water sources [3]. Several procedures and tools have been developed for assessing these contaminants. This may involve different parameters such as conductivity, total suspended solids (TSS), turbidity, total dissolved solids (TDS), and heavy metals. If these factors are in higher concentrations than the limits set by the World Health Organization (WHO) and other regulatory entities, they might affect the quality of drinking water [4].

According to the WHO, pH has no direct influence on water quality; however, it may alter the degree of corrosion of metals and disinfection process efficiency. Thus, an adverse impact on human health may result from the increased digestion of metals from pipes or an ineffective disinfection process.

Pure water is not a conductor of electric current. Generally, the amount of total dissolved solids (TDS) in water determines the electrical conductivity (EC) [5]. The Environmental Protection Agency (EPA) states that conductivity increases when salinity increases. On the other hand, organic composites such as oil are known to be very weak in conducting electrical current and have a low conductivity when mixed in water. Conductivity is also influenced by temperature; as the water heats up, its conductivity increases [6]. Hard water is believed to be hazardous to human health. Calcium and magnesium are the two main ions causing water hardness. An excess intake of these ions may increase the risks of osteoporosis, nephrolithiasis, colorectal cancer, hypertension and stroke, coronary artery disease, insulin resistance, and obesity [7].

Dissolved oxygen (DO) and chlorides are the most important factors of water quality. Water quality is low when the DO concentration is low. The DO level in water is affected by salinity and temperature. While it has no direct impact on human health, low concentrations of DO may make water unpalatable to people [8]. Regarding chloride ions, they can cause an extensive variety of biological and environmental impacts on ecosystems. High chloride ion concentrations can lead to salinization, water treatment plant malfunctions, and groundwater contamination. Furthermore, high amounts of chlorides in drinking water may cause gastroenteritis [9]. Consequently, water quality examination is a must worldwide.

In tap water, in addition to heavy metal contamination and other harmful substances, controlling potential microbial contamination is necessary. Therefore, many countries require the use of residual disinfectants in drinking water [10]. The presence of disinfectants can lead to the formation of potentially carcinogenic byproducts, issues with corrosion, and complaints since people dislike the taste of disinfectants in drinking water. In many countries, even when tap water quality is considered excellent, bottled water consumption is increasing. Statistics from low-middle-income countries indicate that bottled water is often contaminated, even if it is safer than tap water [11]. A paper published in 2020 [12] reviewed the current studies on the existence of six emerging contaminants, including microplastics, pharmaceuticals and personal care products, bisphenol A, phthalates, alkylphenols, and perfluoroalkyl and poly-fluoroalkyl substances in bottled water from several countries. The results imply that microplastics within the size range of 1–5 μm are the main and potentially toxic classes of microplastics in bottled water. Moreover, other contaminants were detected at significant levels. The contamination level was also found to be dependent on bottle type. Water in plastic bottles with plastic caps was more polluted than that in glass bottles.

Clay pots can reduce microbial contamination [13]. According to a study conducted in 2004, when comparing water quality between plastic and clay containers, water quality in clay containers was noticeably better than that in plastic containers [14]. Another study in India stated that when using earthenware pots, the *E. coli* count was almost zero at the end of day 3 after water storage [15].

In monetary terms, bottled water prices are hundreds of times higher per liter than tap water prices. As a result, low- and middle-income families tend to drink less bottled water [16]. Moreover, the life cycle of bottled water has a significant impact on climate change compared with tap water, as it produces CO_2_ 180 times more than tap water [17]. Accordingly, using clay pots is considered a sustainable practice. Since clay pots are made from clay, they decompose naturally without polluting the environment. Moreover, they can be used many times without fear of contamination as they only need to be washed and disinfected by drying them in the oven.

In Egypt, many studies have investigated the use of clay pots as a drinking water storage vessel and also the use of clay as a filter to purify drinking water before usage. However, as far as we know, there are no research papers that discuss the drinking water quality after using clay pots as a storage vessel in Egypt. This work aimed to study the use of clay pots as storage containers for tap water and its effect on drinking water quality criteria as well as their use as a post-treatment method for tap water. This may provide an option for reducing the need for bottled water and increasing the use of tap water with the help of clay pots, as a sustainable practice.

## Methods

### Study design

**Fig. 1:**
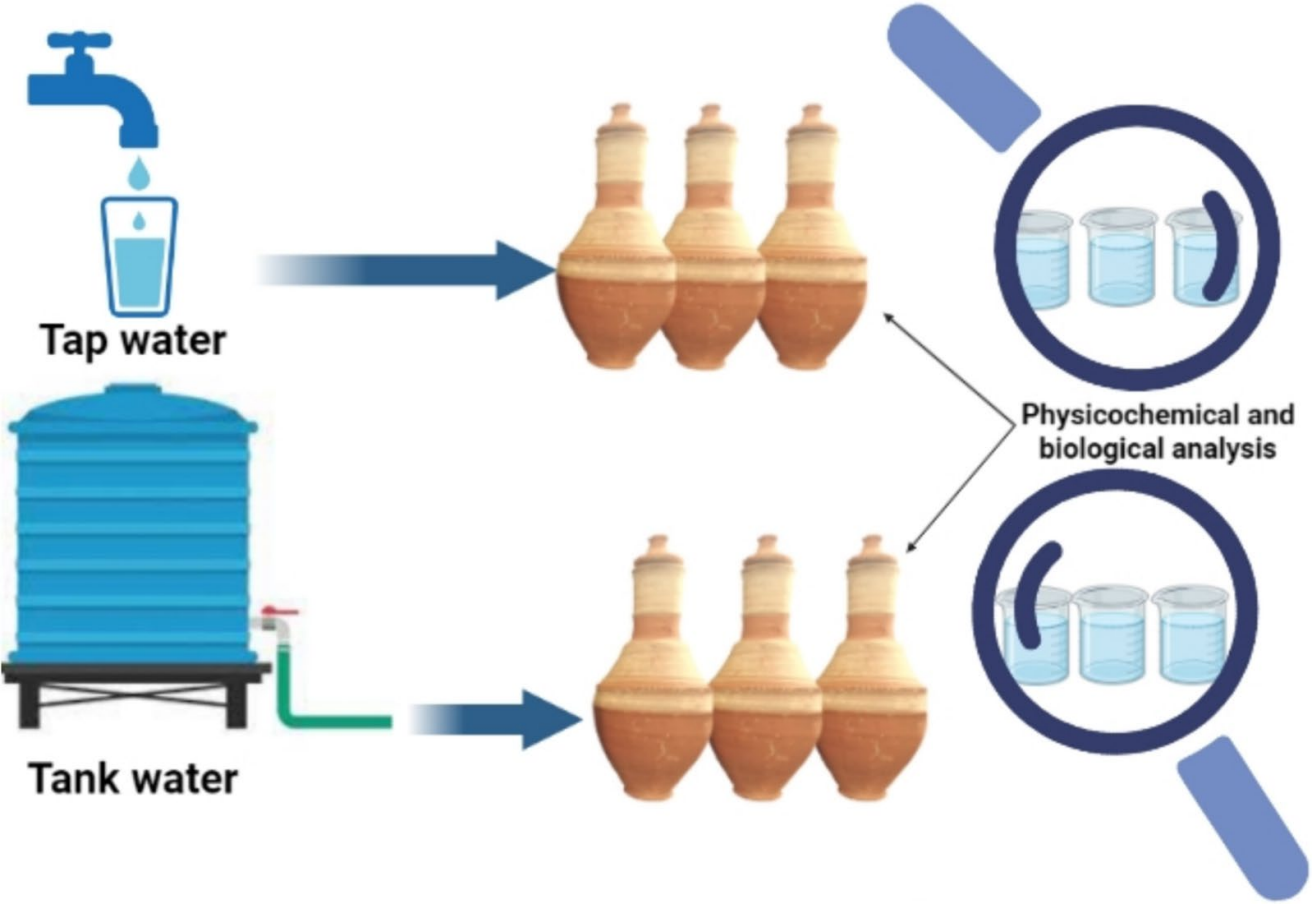
Experimental design

The experimental design in this study is illustrated in Fig. 1. The samples were collected using the grab sampling technique, from tap water and tank water of the Environmental Health Department laboratory at the High Institute of Public Health during the working day at a normal water flow rate.

### Sample size

According to the standard methods for the examination of water and wastewater [18], a total number of 18 water samples were divided into two groups (tap water and tank water). Each group contained nine samples before and after treatment.

### Sampling technique

According to the standard methods for the examination of water and wastewater [18], a total of 1.8 L of water samples (100 ml “maximum” of each sample daily for 7 days) were collected from the clay pots of both tap water and tank water.

### Description of the study materials: clay pot preparation

According to the standard methods for the examination of water and wastewater, six clay pots were used, three for storing tap water, and the other three for tank water storage [18]. The six clay pots were dried in an oven at 180 °C for approximately 2 h. Three clay pots were washed three times with tap water, soaked in tap water for 24 h, and then filled with tap water (group 1). The remaining clay pots were washed three times with tank water, soaked in tank water for 24 h, and then filled with tank water (group 2). All six clay pots were covered with clay lids to ensure no contamination. Samples were collected by pouring the pots into beakers washed with distilled water for analysis.

### Sampling methods and water analysis

#### Water analysis before using clay pots

Tap water and tank water samples were taken according to the sampling technique specified in the standard method for water and wastewater [18]. The following parameters were tested for the samples: pH, EC (electrical conductivity), TDS (total dissolved solids), hardness, D.O. (dissolved oxygen), temperature, chlorides, ammonia, *Legionella*, and TPC (total plate count).

#### Water analysis from clay pots during and after 7 days

The clay pots were filled and left for 7 days. During the experiment (7 days), samples were taken and analyzed for only pH, TPC, D.O., temperature, TDS, hardness, and nitrate [18] to monitor the changes in the water quality during the 7 days. After the 7 days, all parameters were tested (as 2.5.1) [18]. Chloride and Zn were examined for the initial and final water samples, whereas all other parameters were examined over 7 days. The comparison was done between the parameters before and after the treatment.

### X-ray diffractometry (XRD) analysis of Ca carbonate (CaCO_3_)

X-ray powder diffraction analysis was performed with MeasSrv (D2-208,219)/D2-208,219 X-ray powder diffractometer on finely powdered samples (empty clay pot, clay pot filled with tap water, and clay pot filled with tank water) using Cu Ka radiation (30 kV and 10 mA), Cu tube with 1.54184 [Å] with a scanning speed of 0.99° 2Ɵ/s and Lynxeye detector. The time constant was set at 0.1 s.

### Statistical analysis

The IBM SPSS 25.0 (Statistical Package for Social Sciences, USA) software was used for statistical analysis. All experiments were done in triplicates, and descriptive statistics for different parameters of different groups were expressed as the mean and standard error (SE). Analysis of variance (ANOVA) of the data was conducted for different parameters (pH, temperature, EC, TDS, turbidity, DO, ammonia, total hardness, Ca hardness, Mg hardness, chlorine, and TPC) over 7 days. All the values with *P* < 0.05, *n* = 3, were considered statistically significant. In addition, a paired *t* test for all parameters was performed before and after 7 days. Pearson correlation tests between different parameters (EC, TDS, and turbidity–temperature and DO–total hardness, Ca hardness and Mg hardness–TPC and *Legionella*) were done.

## Results

### Physicochemical parameters

The physicochemical characteristics of the different water samples (tap and tank water) which were stored in clay pot vessels were analyzed, and it was found that the water quality of the vessels varied. The results for EC, temperature, TDS, turbidity, DO, ammonia, total hardness, Ca hardness, magnesium hardness, and chlorine are displayed in Table 1. All mean physicochemical parameters, except turbidity, were within the acceptable limits of the Egyptian law for drinking water quality [19] for the different water samples. For turbidity, tap water was within the acceptable limits (0.7 NTU), whereas tank water had a mean turbidity of 1.86 NTU which is higher than the maximum value.

**Table 1:** Descriptive statistics of physicochemical and biological parameters of tap and tank water samples over 7 days

Parameter	Groups				Egyptian law [19]
	**Tap water**		**Tank water**		
	**Mean**	**SE**	**Mean**	**SE**	
**pH**	8.67	0.26	8.67	0.19	6.5–8.5
**Temperature (°C)**	25.37	0.71	25.27	0.65	–
**EC (μs/cm)**	442.71	50.74	500.50	65.36	2000
**TDS (ppm)**	292.041	19.40	326.67	34.18	1000
**Turbidity (NTU)**	0.70	0.25	1.86	0.39	1
**DO (ppm)**	7.46	0.35	7.49	0.25	-
**Ammonia (ppm)**	0.10	0.029	0.09	0.023	0.5
**Total hardness (ppm)**	181.87	12.56	214.17	13.26	500
**Ca hardness (ppm)**	82.29	16.28	108.33	26.05	350
**Mg hardness (ppm)**	96.45	17.81	115.33	19.02	150
**Chlorine (ppm)**	0.075	0.02	0.073	0.023	5
**TPC**	84.33	78.44	288.04	190.53	< 50 cells/cm^3^

*EC* electrical conductivity, *TDS* total dissolved solids, *DO* dissolved oxygen, *TPC* total plate count

Table 2 shows the variances in these parameters before and after storage. During the 7 days, the pH median was around 8 with a small variation for tap water and tank water. In both tap and tank water samples, the temperature of the samples decreased by approximately 2 or 3 °C. TDS, turbidity, ammonia, and chlorine levels also declined over time. The comment on the changes in TPC and *Legionella* will be mentioned in the section on biological parameters.

**Table 2:** Mean of physicochemical and biological parameters of tap and tank water samples before and after 7 days

Groups	Parameters	Mean		Egyptian law [19]
		**Before**	**After**	
	**pH**	8.16	8.59	6.5–8.5
**Tap water**	**Temperature (°C)**	27.00	24.00	-
	**EC (μs/cm)**	542.00	270.00	2000
	**TDS (ppm)**	338.00	275.33	1000
	**Turbidity (NTU)**	1.76	0.84	1
	**DO (ppm)**	6.17	7.52	-
	**Ammonia (ppm)**	0.13	0.074	0.5
	**Total hardness (ppm)**	195.00	178.33	500
	**Ca hardness (ppm)**	85.00	70.00	350
	**Mg hardness (ppm)**	110.00	83.33	150
	**Chlorine (ppm)**	0.09	0.0433	5
	**Chloride (ppm)**	60.00	73.33	250
	**Zinc (ppm)**	0.045	0.12	3
	**TPC**	174.33	2	< 50 cells/cm^3^
	***Legionella***	42.00	2	-
	**pH**	8.31	8.76	6.5–8.5
**Tank water**	**Temperature (°C)**	26.17	24.00	-
	**EC (μs/cm)**	531.67	314.67	2000
	**TDS (ppm)**	324.33	298.00	1000
	**Turbidity (NTU)**	2.19	1.35	1
	**DO (ppm)**	6.5	7.32	-
	**Ammonia (ppm)**	0.13	0.076	0.5
	**Total hardness (ppm)**	201.67	226.67	500
	**Ca hardness (ppm)**	105.00	95.00	350
	**Mg hardness (ppm)**	96.67	131.67	150
	**Chlorine (ppm)**	0.11	0.02	5
	**Chloride (ppm)**	55.00	62.77	250
	**Zinc (ppm)**	0.085	0.14	3
	**TPC**	334	30.00	< 50 cells/cm^3^
	***Legionella***	13	1.67	-

*EC* electrical conductivity, *TDS* total dissolved solids, *DO* dissolved oxygen, *TPC* total plate count

Independent samples *T* test showed that there was a significant difference between the different types of water samples in the physicochemical parameters (*p* equal 0.016, 0.000, 0.000, 0.021, 0.041, 0.00213, and 0.009 for TDS, turbidity, TH, CaH, MgH, chlorine, TPC respectively), based on the *p*-value with ≥ 95% confidence level between different types of water samples. However, pH, temperature, EC, DO, chloride, ammonia, and Zn levels were almost similar in the different water samples (untabulated data) (Table 3).

**Table 3:** Independent samples *T* test of physicochemical parameters and TPC versus tap and tank water samples

	**Tap water**		**Tank water**		
**Parameters**	***M***^**a**^	***SE***^**b**^	***M***^**a**^	***SE***^**b**^	***P***^**c**^
pH	8.67	0.05	8.67	0.04	0.99
Temperature	25.37	0.25	25.27	0.23	0.763
EC	442.7	17.95	500.5	23.12	0.054
TDS	292.04	6.87	326.67	12.08	0.016
Turbidity	0.70	0.09	1.86	0.14	0.000
DO	7.46	0.13	7.5	0.09	0.799
Chloride	16.67	6.1	14.72	5.43	0.813
Ammonia	0.1	0.01	0.09	0.008	0.57
TH	181.87	4.4	214.17	4.69	0.000
Ca-H	82.29	5.75	108.33	9.21	0.021
Mg-H	96.45	6.29	115.83	6.72	0.041
Chlorine	0.075	0.007	0.07	0.008	0.00213
Zn	0.02	0.008	0.028	0.01	0.59
TPC	84.83	27.73	288.04	67.5	0.009

^a^Mean

^b^Standard error

^c^Probability

*EC* electrical conductivity, *TDS* total dissolved solids, *DO* dissolved oxygen, *TH* total hardness, *TPC* total plate count

To test the null hypothesis that the physicochemical parameters (EC, TDS, turbidity, DO, chloride, ammonia, chlorine, TH, CaH, MgH, and Zn) before and after 7 days were equal, a dependent samples *t* test was performed. Prior to conducting the analysis, an assumption of normally distributed differences in readings was made.

For tap water, the null hypothesis of equality of the before and after data means of EC, TDS, turbidity, and DO was rejected, *t*(2) = 20.14, 4.93, 19.07, and 5.47, respectively (*p* < 0.05). For tank water, the null hypothesis of equality of the before and after data means of EC, DO, and chlorine was also rejected, *t*(2) = 22.03, 4.50, and 10.58, respectively (*p* < 0.05). The null hypothesis of equality before and after data mean of Zn for both water samples (tap water and tank water) was rejected *t*(2) = 13.05 and 31.34, respectively (*p* < 0.05) (Table 4).

**Table 4:** Paired *t* test of physicochemical and biological parameters before and after 7 days

**Sample**	**Parameters**	**Before**		**After**		*p*-value
		***M***	***SE***	***M***	***SE***	
Tap water	EC (μc/cm)	542.00	0.58	270.00	13.00	0.002
	TDS (ppm)	338.00	3.00	275.33	14.65	0.039
	Turbidity (NTU)	1.75	0.067	0.84	0.021	0.003
	DO (ppm)	6.17	0.09	7.5	0.16	0.032
	Chloride (ppm)	60.00	2.89	73.33	6.00	0.094
	Ammonia (ppm)	0.13	0.005	0.074	0.011	0.075
	Chlorine (ppm)	0.093	0.017	0.043	0.018	0.138
	TH (ppm)	195.00	2.89	178.33	13.64	0.267
	Ca-H (ppm)	85.00	5.77	70.00	7.64	0.225
	Mg-H (ppm)	110.00	7.64	83.33	14.81	0.347
	Zn (ppm)	0.045	0.000	0.12	0.006	0.006
	TPC	174.33	162.9	2.00	1.5	0.045
	*Legionella*	42.0	25.15	2.0	2.0	0.002
Tank water	EC (μc/cm)	531.67	6.56	324.67	15.89	0.002
	TDS (ppm)	324.33	6.33	298.00	13.00	0.104
	Turbidity (NTU)	2.19	0.35	1.35	0.031	0.161
	DO (ppm)	6.53	0.17	7.32	0.13	0.046
	Chloride (ppm)	55.00	2.89	62.76	9.25	0.463
	Ammonia (ppm)	0.13	0.009	0.076	0.017	0.095
	Chlorine (ppm)	0.11	0.003	0.02 ±	0.017	0.009
	TH (ppm)	201.67	6.00	226.66	17.4	0.392
	Ca-H (ppm)	105.00	2.89	95.00	7.64	0.184
	Mg-H (ppm)	96.67	7.26	131.67	12.02	0.206
	Zn (ppm)	0.085	0.00	0.14	0.0017	0.001
	TPC	334.00	166.00	30.00	11.55	0.001
	*Legionella*	13.00	7.5	1.67	1.67	0.025

*M* mean, *SE* standard error, *p* probability, *EC* electrical conductivity, *TDS* total dissolved solids, *DO* dissolved oxygen, *TH* total hardness, *TPC* total plate count

Thus, a significant difference was found between the samples taken before and after 7 days for both settings in some parameters, indicating that the clay pot was effective in enhancing drinking water quality.

### Biological parameters

The TPCs of the different water samples (tap water and tank water) were assessed in clay pot vessels. The study found that different water samples had varying water quality. The TPC means of tap water and tank water samples (Table 1) exceeded the acceptable limits of Egyptian law for drinking water quality [19]. The lower concentration of TPC and *Legionella* in the water after storing it in clay pots for 7 days was highly noticeable (Table 2).

To test the null hypothesis that the biological parameters (TPC and *Legionella*) before and after 7 days were equal, a dependent samples *t* test was performed. Before conducting the analysis, an assumption of normally distributed differences in readings was made.

For the different types of water samples, the null hypothesis of equality before and after data means of TPC was rejected, *t*(2) = 4.54 and 40.70, for tap water and tank water, respectively; for *Legionella*, it was rejected, *t*(2) = 20.18 and 6.19, for tap water and tank water, respectively (*p* < 0.05) (Table 4). Thus, there was a significant difference in TPC and *Legionella* before and after 7 days for the different water samples, indicating that the clay pot was effective in removing these pathogens to enhance the drinking water quality.

### Correlation between different parameters

The correlation matrix between the EC, TDS, and turbidity showed a strong positive correlation. According to the Pearson correlation, the correlation (*r*) between EC and TDS, EC and turbidity, and TDS and turbidity were highly significant (*r* = 0.976, 0.717, and 0.708, respectively (*p* < 0.01) (Table 5), corresponding to a simultaneously increasing relationship between these parameters.

**Table 5:** Pearson correlation between EC, TDS, and turbidity between tap and tank water samples

Parameters	EC	TDS	Turbidity
EC	1	0.976^a^	0.717^a^
TDS		1	0.708^a^
Turbidity			1
**Parameters**	**Temperature**		**DO**
Temperature	1		0.978^a^
DO			1
**Parameters**	**TH**	**Ca_H**	**Mg_H**
TH	1	0.818^a^	0.898^a^
Ca_H		1	0.660^a^
Mg_H			1
**Parameters**	**TPC**	***Legionella***	
TPC	1	0.344	
*Legionella*		1	

^a^Correlation is significant at the 0.01 level (2-tailed)

*EC* electrical conductivity, *TDS* total dissolved solids, *DO* dissolved oxygen, *TH* total hardness, *Ca_H* calcium hardness, *Mg_H* magnesium hardness, *TPC* total plate count

The correlation matrix between temperature and DO indicated a strong positive correlation. According to the Pearson correlation, the correlation (*r*) between temperature and DO was highly significant (*r* = 0.978 (*p* < 0.01) (Table 5), indicating a simultaneously increasing relationship between these parameters.

The correlation matrix between TH, Ca_H, and Mg_H showed a strong direct correlation. According to Pearson correlation, the correlation (*r*) between TH and Ca_H, TH and Mg_H, and Ca_H and Mg_H were highly significant (*r* = 0.818, 0.898, and 0.660, respectively (*p* < 0.01) (Table 5), which indicated the simultaneously increasing association between these parameters as TH equals the sum of Ca_H and Mg_H.

The correlation matrix between TPC and *Legionella* showed a very weak direct correlation. According to Pearson correlation, the correlation (*r*) between TPC and *Legionella* was not significant, *r* = 0.344 (*p* < 0.05) (Table 5).

**Fig. 2:**
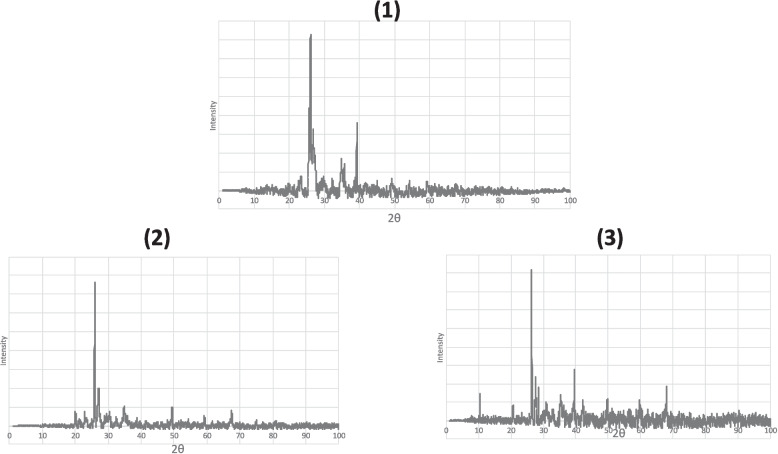
XRD of CaCO_3_. (**1**) Empty clay pot. (**2**) Clay pot filled with tap water. (**3**) Clay pot filled with tank water

As shown in Fig. 2, XRD peaks of the characteristic Ca carbonate (CaCO_3_) appeared sharp, clearly distinct, and broad, which confirms the existence of CaCO_3_ compounds as a main component in the clay pot structure. The XRD pattern displayed characteristic peaks of CaCO_3_, which were at almost the same 2Ɵ = 104° for all samples (empty clay pot, clay pot filled with tap water, and clay pot filled with tank water).

## Discussion

The water quality of in storage containers (clay pots) may be impacted if it is directly or indirectly exposed to external factors such as temperature, wind, and dust. Household water quality is affected by the cleaning of water storage tanks and vessels. Water storage is globally observed and may reflect a lack of trust in the government-run water supply infrastructure. Water storage tanks can be cleaned manually with specialized mops or brooms, mechanical scrubbers, water jets, hoover cleaners, and special chemicals [20].

Several studies have investigated tank cleaning. Sule et al. [21] noted that inadequate hygiene methods and dubious source water contributed to a significant decline in the quality of stored water in Ilorin, Nigeria. In another study, a relationship was established between tank handling procedures in Zaria, Nigeria, and the quality of stored water [22]. In South Africa, houses were supplied by water tankers, so it was safer to clean the tank before each filling to ensure the quality of the water from tanker trucks and prevent the transfer of pollutants from the previous supply [23].

For this study, the physicochemical parameters pH, temperature, EC, TDS, turbidity, total hardness and its fractions, ammonia, chlorine, chloride, and Zn were tested. The pH of tap water and tank water samples (8.75 and 8.69, respectively) in this study was slightly higher than the pH value in another study which also used clay pots (7.9, 7.8, and 7.5, respectively) [24]. The pH increases after storage may be due to the drop in aqueous carbon dioxide (CO_2_) in the stored water since the clay pot is alkaline in nature, which increases the pH of water [25]. The pH of water is influenced by several factors, including the water source, the material of the water storage tank or vessel, temperature, mineral absorption, dust, amount of bacterial activity in the vessel, and amount of time the water is stored until usage [26]. The alkaline composition of clay is another advantage of clay waterpots. The correct pH balance is produced by the interaction between the alkaline clay and the acidity of the water. This water helps relieve gastrointestinal discomfort by reducing acidity [27].

According to Verploegen et al. [28], water evaporating through the pores of an earthen vessel keeps the remaining water inside cold. This might be the reason why the temperature of the water samples (both tap water and tank water)) kept in the clay pot vessel decreased to 24 °C at the end of the 7 days in the current study. The earthen pot’s particular property, which no other container possesses, is that it transfers coolness to the water according to the weather [27]. Compared with plastic containers, clay pots are more frequently used in the rural community to store water across all houses. This can be due to the affordability of plastic containers, the cost of making clay pots, or the fact that clay pots help in lowering the water temperature [29].

In the present study, for tap water and tank water samples in clay pots, the TDS of each water sample was found to be quite low in comparison to Egyptian law [19]. TDS in tap water kept in clay pots significantly decreased (*p* < 0.05), while TDS in tank water also kept in clay pots was not substantially altered (*p* > 0.05), which explains the significant difference between tap and tank water. The findings for tap water and tank water were compatible with the results of calabash clay pot vessels and other vessels, respectively [30]. Another research using clay pots had lower observations (64.4, 70.1, and 88.5 mg/L) [24] than this study’s results, which might be because the original TDS of our study’s samples was very high (338 and 324.33 mg/L for tap water and tank water, respectively). The elevated TDS of water samples in clay pots may be attributed to an increase in the mineral quantity, influenced by the properties of clay compared to other materials for containers, such as steel vessels or plastic bottles. The decreased TDS observed later could be due to the settling down of the minerals present in the water [25].

Electrical conductivity is an indirect indicator of dissolved inorganic particles because it measures the extent to which water can conduct an electric current. This aids in determining whether water is suitable for domestic and agricultural usage [25]. The EC values in the current study for tap water and tank water, ranged between 257–546 and 288–881 s/cm, respectively, which was greater than the finding of Neethu et al. [25], which was 117.3–154.8 s/cm. There was a significant difference in EC values between tap water and tank water samples, and there was a distinctive significant difference in the initial EC and the final EC in both tap water and tank water samples. This is consistent with the results of a study in Nigeria which showed a significant difference in EC for water stored in a clay pot (47.67 μs/cm) in comparison with the original value of EC (50.57 μs/cm) [31].

The findings of the present study showed that there was a significant difference in turbidity between tap water and tank water samples, but only tap water samples had a significant difference between the initial value and the end value, while tank water samples had no considerable differences. In this study, the reduced turbidity observed in clay pot-stored water may reduce vessel bacteria, protozoa, and helminths as well as improve taste and smell [30]. The turbidity removal in this study was higher than that observed by Obianyo et al. (2020 (6.33 NTU) where turbidity of water showed a slight but significant variation after storage in clay pots [31]. This decrease in the turbidity value of water after placing it in a clay pot container is a sign that the water quality has improved.

In the present study, the DO of each water sample stored in the clay pot was found to be high (7.46 and 7.49 mg/L for tap water and tank water samples, respectively), which accounts for the lack of statistical significance between the two types of water samples (*p* > 0.05). However, there was a significant difference between the two time periods (before and after storage) (*p* < 0.05). In another study, it was reported that the DO of each water sample held in a clay pot was high (7.25 mg/L, 6.75 mg/L, and 7.1 mg/L, respectively) which is consistent with our study’s results [24]. In addition, DO in the water samples (tap water and tank water) increased significantly (*p* < 0.05) in clay pots, which is consistent with the findings of research using calabash and clay pots [30]; however, there were no significant effects on DO for water held plastic and metal vessels (*p* > 0.05). In our study, there was a significant difference between tap and tank water samples.

In the current investigation, the chloride concentrations of tap and tank water samples were not substantially influenced by their storage in clay pots (*p* < 0.05), and these results are consistent with Duru et al. [30].

The recommended level of ammonia according to Egyptian drinking water guidelines is 0.5 mg/L [19], which is consistent with the findings of the present study. In this study, there was no significant difference found in ammonia levels between clay pots filled with tap water and those filled with tank water, and there was no significant difference before and after 7 days of storage in both tap water and tank water samples, which confirms that there was no significant conversion of ammonia to nitrate. Nitrate and nitrite in drinking water are thought to induce cancer in the gastrointestinal and urinary tracts, as well as at other locations, because they function as precursors of genotoxic N-nitroso compounds in endogenous nitrosation [32].

Carbonates, bicarbonates, sulfates, Ca and magnesium chloride, and chlorides are the main causes of drinking water hardness [33]. The present study indicated that the hardness and its fractions (Ca hardness and Mg hardness) of each stored water sample showed no significant differences between the initial and final values. There was a significant difference (*p* < 0.05) in the total hardness and its fractions (Ca hardness and Mg hardness) between the two types of water samples (tap water and tank water). The hardness of both water samples was slightly higher than the results of a study using clay pots (200, 200, and 150 ppm, respectively) [24]. Since the clay or minerals used to make the pots include hardness-containing ions, the total hardness of the water in the pot increases [34].

Chlorine is widely used as a disinfectant, particularly in underdeveloped nations such as Egypt. One of the most important steps in stopping the spread of potentially fatal waterborne infections is chlorine disinfection [35]. There are various causes for disinfectant depletion, which can have an impact on their effectiveness. Disinfectants react readily because they are strong electron acceptors and oxidizing agents. For instance, depletion of disinfectants may result from a biofilm developing in the water distribution system. In addition to photodegradation, pH, and temperature, other factors can contribute to disinfectant depletion which affects their effectiveness [36].

Consequently, in the present investigations, the TPC declined over time while the chlorine concentration had no significant difference before and after 7 days for tap water, while tank water’s chlorine concentration showed a significant difference before and after 7 days, which might be because the higher TPC in tank water compared to tap water caused the consumption of chlorine with the TPC of tank water. Chlorine reacts with both inorganic and organic substances (such as metals and humic and fulvic acids) when added to water, rendering it unsuitable for disinfection. The amount of chlorine consumed in these reactions is known as the chlorine demand of water and is calculated empirically. The remaining chlorine is known as the total chlorine residual (TCR) once the chlorine requirement is satisfied. Chlorine residuals are thought to completely prevent biofilm accumulation, which lowers the dangers connected with biofilm for water quality and public health (such as discoloration and any related microbiological mobilization). This belief is still prevalent in the water industry and the general public [37].

Zinc (Zn) concentrations above 3.0 mg/L in drinking water might give water an unpleasant metallic flavor [19, 38]. The results of this study showed a significant increase in Zn concentration from the beginning to the end, but it was within the acceptable limit [19]. Zn is less harmful to human health, and its deficiency may affect immunological function, growth, and neural development in humans [39].

Based on the results, clay pots proved their efficiency in decreasing some physicochemical parameters (EC, turbidity, ammonia, TDS, TPC, and *Legionella*) and increasing others (DO, chloride, and Zn). In clay manufacturing, clay is combined with water and organic components and burnt at a high temperature. It develops porosity, mechanical strength, and chemical stability or inertness [40]. Most clays remain robust even after burning at temperatures exceeding 1000 °C. To achieve temperatures above 1000 °C, a kiln must be built to contain the heat [41] which contributes to its durability. As a result, the structure has pores that are both big enough to let water flow through and small enough to keep out bacteria and other impurities. This method of making clay pots should be able to filter out all particles and bacteria bigger than the pore size [13].

The composition of the clay, firing temperature, particle size, ramming (manual or hand consolidation/forming) pressure, additives, and the reaction that takes place during the manufacturing all affect the mechanism of action of these pots, the water discharge rate, and microbe elimination efficiency [42]. Adsorbents made of clay minerals include kaolinite and bentonite. Kaolin has a low expansion coefficient, superior cation exchange capacity, and great chemical stability [43]. Because of its superior cation exchange capacity, kaolinite performs well when it comes to removing ions from aqueous solutions [44]. A clay pot can be used as a cooling system to lower the temperature whereby it simultaneously raises the relative humidity inside the inner clay pot and evaporates water off the outer surface of the clay pot [45]. This action mechanism of clay explains its ability to disinfect and remove pollutants.

Pontiac fever and Legionnaires’ disease transmission are thought to be possible in water contaminated with *Legionella* spp. It is crucial to regularly monitor hospital water supplies for the pathogen to prevent outbreaks of *L. pneumophila*, especially in hospitalized patients with impaired immune systems. There is an ongoing discussion on the relationship between the presence of *L. pneumophila* in water samples and the danger of infection to people’s health. An investigation in the United States has established a connection between Legionnaires’ disease outbreaks in the supplied areas and the presence of the organism in drinking water samples [46]. According to the present study’s findings, there was a significant drop in the concentration of *Legionella* from the beginning to the end in both water samples, which proved the efficiency of the clay pot in its removal.

The correlation between different parameters was examined. There was a strong correlation between EC, TDS, turbidity, temperature, DO, and total hardness and its fractions (Ca hardness and Mg hardness), while there was a weak correlation between TPC and *Legionella*.

Of the studies included in a research article, electrical conductivity (EC) was the second most frequently used indicator of water quality. Although there was a link between the TDS level and EC, 10 of the 24 (42%) investigations used EC as a dependent variable. This was demonstrated in the study by Akuffo et al. [47], where the EC value increased in correlation with the TDS value. According to standard methods for water and wastewater analysis [18], the conductivity of water is influenced by the dissolved solids present. Since the EC and TDS of water are interdependent, as TDS drops in the clay pot, EC declines as well, and vice versa [48]. The findings of the present study are consistent with the significant correlation between TDS and EC. In addition, because conductivity measures how well water can carry an electrical current, it is correlated with the ionic composition of the water. This indicator is useful because sudden or severe fluctuations at the organism level can indicate issues with the water supply. The water must be “acceptable to consumers and [there must be] no abnormal change” to meet the parametric value for turbidity (at the tap water) (1.0 NTU). Nonetheless, it must be emphasized that this value refers to how well the water looks [6]. This was consistent with the correlation between TDS and turbidity.

The water directly contacts the air in clay pots, increasing the DO of the water, which explains the negative correlation between temperature and DO [48]. According to published data, the rate of calcite dissolution increases sharply from pH 4 to 1, but it is rather flat across the center of the pH range before declining slightly once more at high pH [49], which is compatible with the research results as the pH was approximately 8, and there was no significant difference in the CaCO_3_ concentration. In addition, this explained the non-significant difference in the total hardness and its fractions before and after 7 days.

Finally, earthen pots can cool liquids without using energy. They biodegrade naturally into the Earth but can also be recycled and reused. Earthen pots are environmentally beneficial owing to their esthetic value. The proposed design concept offers a method for natural water filtering and purification. Doing this at home is both simple and affordable [27].

### Limitations

Two major limitations in this study that could be addressed in future research. First, the study focused on one type of clay pots which were purchased from a random seller. Second, there was a lack of previous research studies on this topic. Regarding the first limitation, due to some financial limitations, the team was not able to study many types of clay pots and investigate the different soil structures that those clay pots were made of or even the process of producing the clay pots. The team believes that this may have a significant impact on the way the clay pot reacts as a method of water treatment. Therefore, it is recommended that further studies explore this aspect in greater detail. Furthermore, financial limitations made the research team able to experiment for only 7 days, and this hindered them from making longer-term observations of the water quality.

As for the second limitation, the team found that there was not enough data and studies on the use of clay pots for storing drinking water; hence, this study aims at only exploring the effectiveness of using clay pots without deeply studying the causes and the consequences of using them. Most of the studies discussed the use of clay as a filter for drinking water treatment. Therefore, further research should be done to truly confirm the efficacy of using clay pots economically and sustainably and understand in depth how clay pots change the quality of drinking water.

## Conclusion

The material of the storage container has a considerable impact on the quality of water. In the clay pot, TDS, EC, turbidity, pH, and DO of the water were altered. As a traditional water storage method, clay pots are inexpensive and highly efficient. This is not similar to sophisticated and expensive water purification techniques. Furthermore, according to the market price in Egypt, although the price of one clay pot is almost double the price of one plastic bottle of water, clay pots can be reused several times after cleaning and disinfection (as sustainable containers) and will not be adversely affected by hot temperatures as what happens in plastic bottles. The fact that it is accessible to everyone, including those in remote places, is what matters the most. Therefore, these could be considered good candidates for water storage.

## Data Availability

All data generated or analyzed during this study are included in this article. In addition, the related datasets are available from the corresponding author upon reasonable request.

## References

[1] Li P, Wu J (2019). Drinking water quality and public health. Expo Health.

[2] Daud M, Nafees M, Ali S, Rizwan M, Bajwa RA, Shakoor MB, et al. Drinking water quality status and contamination in Pakistan. BioMed Res Int. 2017;2017. 10.1155/2017/7908183 10.1155/2017/7908183 PMC5573092 28884130

[3] Mohsin M, Safdar S, Asghar F, Jamal F. Assessment of drinking water quality and its impact on residents health in Bahawalpur city. Int J Humanit Soc Sci. 2013;3(15):114–28. http://www.ijhssnet.com/journals/Vol_3_No_15_August_2013/14.pdf.

[4] Rahmanian N, Ali SHB, Homayoonfard M, Ali N, Rehan M, Sadef Y (2015). Analysis of physiochemical parameters to evaluate the drinking water quality in the State of Perak. Malaysia J Chem.

[5] WHO. Guidelines for drinking-water quality, 4^th^ edition. World Health Organization. 2017; 631 pp . Available from: https://www.who.int/publications/i/item/9789241549950. Accessed 24 Apr 2023.

[6] EPA I. Drinking water parameters microbiological, chemical, and indicator parameters in the 2014 drinking water regulations: johnstown castle estate wexford. Ireland; 2014. Available from: https://www.epa.ie/publications/compliance--enforcement/drinking-water/Drinking-Water-Report-2014_Final.pdf. Accessed 30 June 2021.

[7] Akram S, Rehman F. Hardness in drinking water, its sources, its effects on humans and its household treatment. J Chem Appl. 2018;4(1):1–4. https://www.avensonline.org/fulltextarticles/JCAP-2380-5021-04-0009.html.

[8] Summers JK, Editor. Water quality: science, assessments and policy. London, United Kingdom: IntechOpen; 2020.pp: 182. 10.5772/intechopen.77531.

[9] Moradi H, Sabbaghi S, Mirbagheri NS, Chen P, Rasouli K, Kamyab H (2023). Removal of chloride ion from drinking water using Ag NPs-Modified bentonite: characterization and optimization of effective parameters by response surface methodology-central composite design. Environ Res.

[10] Rosario-Ortiz F, Rose J, Speight V, Gunten UV, Schnoor J (2016). How do you like your tap water?. Science.

[11] Williams AR, Bain RE, Fisher MB, Cronk R, Kelly ER, Bartram J (2015). A systematic review and meta-analysis of fecal contamination and inadequate treatment of packaged water. PLoS ONE.

[12] Akhbarizadeh R, Dobaradaran S, Schmidt TC, Nabipour I, Spitz J (2020). Worldwide bottled water occurrence of emerging contaminants: a review of the recent scientific literature. J Hazard Mater.

[13] Varkey A, Dlamini M (2012). Point-of-use water purification using clay pot water filters and copper mesh. Water Sa.

[14] Trevett AF, Carter RC, Tyrrel SF (2004). Water quality deterioration: a study of household drinking water quality in rural Honduras. Int J Environ Health Res.

[15] Brick T, Primrose B, Chandrasekhar R, Roy S, Muliyil J, Kang G (2004). Water contamination in urban south India: household storage practices and their implications for water safety and enteric infections. Int J Hyg Environ Health.

[16] Bain R, Cronk R, Wright J, Yang H, Slaymaker T, Bartram J (2014). Fecal contamination of drinking water in low-and middle-income countries: a systematic review and meta-analysis. PLoS Med.

[17] Cohen A, Ray I (2018). The global risks of increasing reliance on bottled water. Nature Sustainability.

[18] Baird RB, Eaton AD, Rice EW. Standard methods for the examination of water and wastewater. 23rd Ed. Washington DC, USA: APHA, AWWA, WEF; 2017. Available from: https://yabesh.ir/wp-content/uploads/2018/02/Standard-Methods-23rd-Perv.pdf.

[19] Ministry of Health and Population. The Ministry of Health decree regarding the maximum limits and specifications needed for drinking water and domestic use. Egypt: Ministry of Health and Population; Resolution No. 458, 2007. Available from: https://www.fao.org/faolex/results/details/en/c/LEX-FAOC083626/.

[20] Nnaji C, Nnaji I, Ekwule R (2019). Storage-induced deterioration of domestic water quality. J Water Sanit Hyg for Dev.

[21] Sule I, Agbabiaka T, Akomolafe A (2011). Bacteriological quality of water stored exteriorly in storage tanks. Res J Environ Sci.

[22] Chia M, Oniye SJ, Swanta AA. Domestic water quality assessment: microalgal and cyanobacterial contamination of stored water in plastic tanks in Zaria, Nigeria. Eur J Sci Res. 2013;110(4):501–10. http://www.europeanjournalofscientificresearch.com.

[23] Singh U, Lutchmanariyan R, Wright J, Knight S, Jackson S, Langmark J (2013). Microbial quality of drinking water from ground tanks and tankers at source and point-of-use in eThekwini Municipality, South Africa, and its relationship to health outcomes. Water Sa.

[24] Kumar P, Garg V, Scholar MT (2020). Effect of different storage vessels on various types of water in KOTA city. Int J Eng Appl Sci Technol.

[25] Mohanan N, Manju E, Jacob S (2017). The effect of different types of storage vessels on water quality. Int J Innovative Res Sci Eng Technol.

[26] Packiyam R, Kananan S, Pachaiyappan S, Narayanan U (2016). Effect of storage containers on coliforms in household drinking water. Int J Curr Microbiol Appl Sci.

[27] Shamaeezadeh M, Zhou H. Proceeding of Cumulus Mumbai 2015: In a Planet of Our Own—a vision of sustainability with a focus on water; 2015 December 3-5; Mumbai, India: IDC, IIT Bombay; 2016. Available from: http://www.inaplanetofourown.net/assets/papers/Massoud%20Shamaeezadeh-%20Cumulus%20Mumbai%202015.pdf.

[28] Verploegen E, Rinker P, Ognakossan K. Evaporative cooling best practices. Massachusetts institute of technology; 2018. Available from: https://www.researchgate.net/publication/325858150_Evaporative_Cooling_Best_Practices_Guide. Accessed 1 Apr 2023.

[29] CDC. Control CFD, Prevention. CDC [National center for emerging and zoonotic infectious diseases (NCEZID), Division of foodborne, waterborne, and environmental diseases (DFWED)]. Reports of E coli Outbreak Investigations from 2018; 2018. Available from: https://www.cdc.gov/ecoli/2018-outbreaks.html. Accessed 20 Nov 2023.

[30] Duru MAC, Amadi B, Nsofor C, Nze H (2013). Effect of different storage vessels on water quality. Global Res J Sci.

[31] Obianyo J. Effect of storage containers on water quality. Trop J Sci Technol. 2020;1(1):66. http://www.credencepressltd.com/journal/uploads/archive/202015934541031130451008.pdf.

[32] Villanueva CM, Kogevinas M, Cordier S, Templeton MR, Vermeulen R, Nuckols JR (2014). Assessing exposure and health consequences of chemicals in drinking water: current state of knowledge and research needs. Environ Health Perspect.

[33] WHO. Water Sanitation and Hygiene: transforming the regional agenda towards equitable access to safe and sustainable services. Copenhagen, Denmark: World Health Organization. 2017. Available from: https://www.who.int/europe/publications/m/item/water--sanitation-and-hygiene. [Accessed 1 October 2023].

[34] Chalchisa D, Megersa M, Beyene A (2018). Assessment of the quality of drinking water in storage tanks and its implication on the safety of urban water supply in developing countries. Environ Syst Res.

[35] Liu H, Zhang X, Fang Y, Fu C, Chen Z (2021). Trade-off control of organic matter and disinfection by-products in the drinking water treatment chain: role of pre-ozonation. Sci Total Environ.

[36] Sharif MO. Towards identifying disinfectants and quantifying disinfectant levels in water. [Master Thesis]. Canada: McMaster University;2017. Available from: https://macsphere.mcmaster.ca/bitstream/11375/22266/2/Sharif_Omar_September2017_MSc.pdf. Accessed 18 Sep 2022.

[37] Fish KE, Reeves-McLaren N, Husband S, Boxall J (2020). Uncharted waters: the unintended impacts of residual chlorine on water quality and biofilms. NPJ Biofilms and Microbiomes.

[38] Plum LM, Rink L, Haase H (2010). The essential toxin: impact of zinc on human health. Int J Environ Res Public Health.

[39] National Health and Medical Research Council (NHMRC). Australian drinking water guidelines paper 6 national water quality management strategy. national health and medical research council, national resource management ministerial council, commonwealth of Australia, Canberra. 2011:5–7. Available from: https://www.lachlan.nsw.gov.au/files/assets/public/v/1/council/australian-drinking-water-standards-2011.pdf

[40] Mominul Alam SM, Kawauchi T, Takeichi T (2010). Preparation and characterization of rigid polyimide-clay-polydimethylsiloxane hybrid. High Perform Polym.

[41] Petersham M, Rhodes D, Rowan G, VITAINC C. Understanding clay recognition and processing: Volunteers in Technical Assistance; 1984. https://pdf.usaid.gov/pdf_docs/PNAAS747.pdf

[42] Lantagne D, Klarman M, Mayer A, Preston K, Napotnik J, Jellison K (2010). Effect of production variables on microbiological removal in locally-produced ceramic filters for household water treatment. Int J Environ Health Res.

[43] Miranda-Trevino JC, Coles CA (2003). Kaolinite properties, structure and influence of metal retention on pH. Appl Clay Sci.

[44] Omar W, Al-Itawi H (2007). Removal of Pb? 2 Ions from aqueous solutions by adsorption on kaolinite clay. Am J Appl Sci.

[45] Verploegen E, Sanogo O, Chagomoka T, editors. Evaluation of low-cost evaporative cooling technologies for improved vegetable storage in Mali. 2018 IEEE Global Humanitarian Technology Conference (GHTC); 2018: IEEE. 10.1109/GHTC.2018.8601894. Accessed 3 Jan 2022.

[46] Beer KD, Gargano JW, Roberts VA, Hill VR, Garrison LE, Kutty PK (2015). Surveillance for waterborne disease outbreaks associated with drinking water—United States, 2011–2012. Morb Mortal Wkly Rep.

[47] Akuffo I, Cobbina S, Alhassan E, Nkoom M. Assessment of the quality of water before and after storage in the Nyankpala community of the Tolon-Kumbungu District, Ghana. Int J Sci Technol Res. 2013; 2(2):221–227. URL: https://www.researchgate.net/publication/258234882_Assessment_Of_The_Quality_Of_Water_Before_And_After_Storage_In_The_Nyankpala_Community_Of_The_Tolon-Kumbungu_District_Ghana

[48] Ogbozige F, Adie D, Ibrahim F. Chemistry of potable water during storage: the northwestern Nigeria perspective. Int J Eng Sci Res. 2015;6(6):549–63. Available from: https://www.ijser.org/researchpaper/CHEMISTRY-OF-POTABLE-WATER-DURING-STORAGE-THE-NORTH-WESTERN-NIGERIA-PERSPECTIVE.pdf.

[49] Arvidson RS, Ertan IE, Amonette JE, Luttge A (2003). Variation in calcite dissolution rates: a fundamental problem?. Geochimica et Cosmochimica Acta [Spanish].

